# Supplementary material for: Generation and Characterization of a Transgenic Mouse That Specifically Expresses the Cre Recombinase in Spermatids
Source: Genes (Basel). 2023 Apr 27;14(5):983. doi: 10.3390/genes14050983 (PMC10217838; doi:10.3390/genes14050983)
Supplement: Supplementary file 1 [file genes-14-00983-s001.zip › genes-2280760-supplementary.pdf]

Supplementary figure legend

Supplementary Figure 1. CRE is expressed in round spermatids from stage V in *Rosa26*$^{mTmG/Acrv1\text{-}iCre}$ males (complement to figure 3).

Representative immunodetection images obtained using antibody against CRE protein on testicular sections from *Rosa26*$^{mTmG/+}$ (used as negative CTL) and *Rosa26*$^{mTmG/Acrv1\text{-}iCre}$ males at different stages. In the merged figure, DAPI is shown in blue, CRE in red an LECTIN in green. DAPI was used to stain the nuclei. In *Rosa26*$^{mTmG/Acrv1\text{-}iCre}$ males, CRE signal starts to be detected in step 5 round spermatids (in stage V tubules) and is stronger in step 6 round spermatids (in stage VI tubules). In step 9 spermatids (stage IX tubule), CRE signal is no more visible. In stage XII-I, no specific signal is observed. Scale bar indicates 20µm.

Supplementary Figure 2. GFP is expressed in elongating spermatids from stage X in *Rosa26*$^{mTmG/Acrv1\text{-}iCre}$ males (complement to figure 3).

Representative immunodetection images obtained using antibody against GFP protein on testicular sections from *Rosa26*$^{mTmG/+}$ (used as negative CTL) and *Rosa26*$^{mTmG/Acrv1\text{-}iCre}$ males at different stages. In the merged figure, DAPI is shown in blue, LECTIN in red and GFP in green. DAPI was used to stain the nuclei. In *Rosa26*$^{mTmG/Acrv1\text{-}iCre}$ males, no specific GFP signal is visible in stage IX tubules. GFP signal starts to be detected in step 10 elongating spermatids (stage X tubules) and is very strong in step 15-16 condensed spermatids (in stage IV to VIII tubules) before they are released in the testis lumen. Scale bar indicates 20µm.

Fig. S1

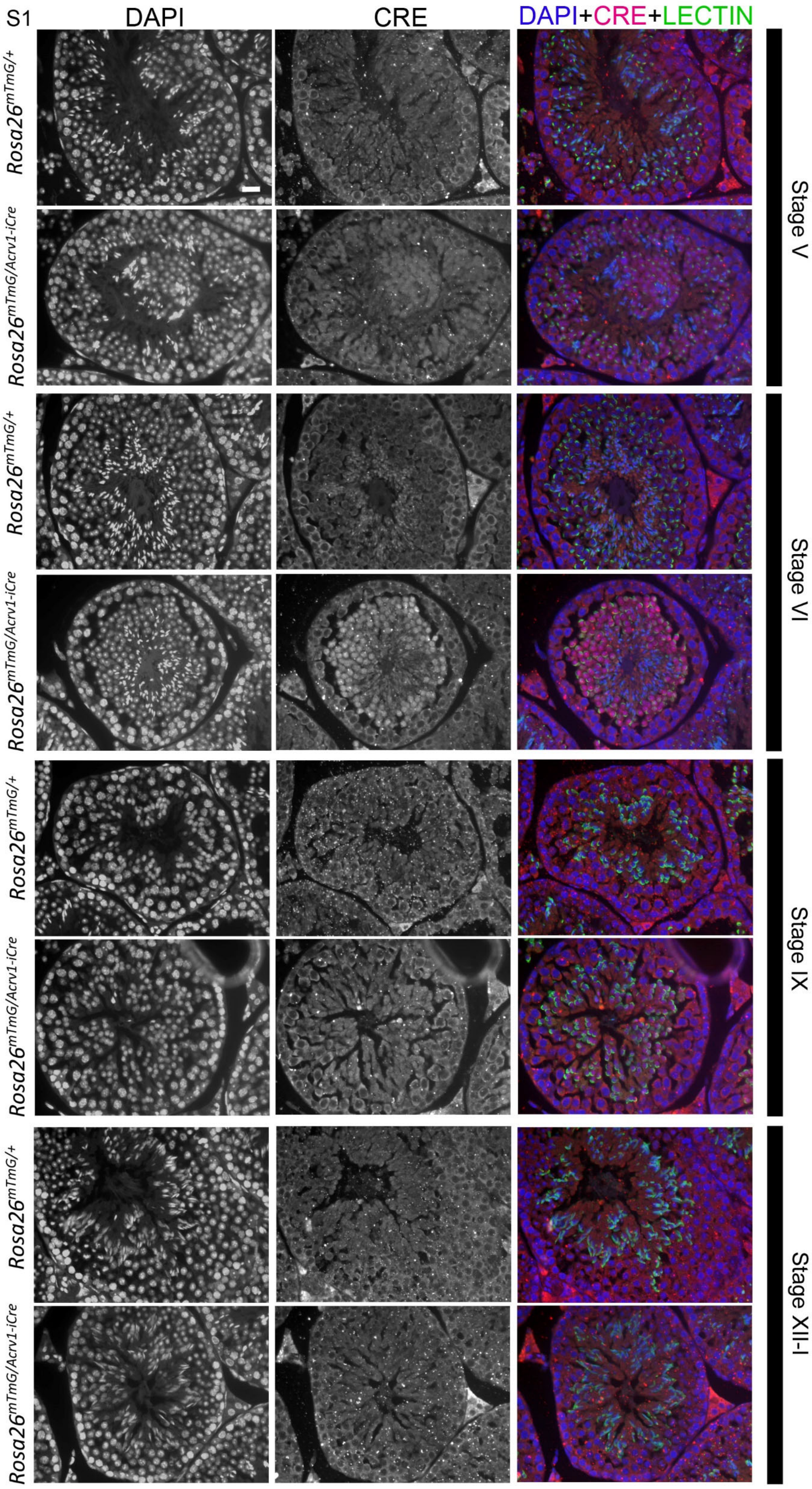
DAPI
CRE
DAPI+CRE+LECTIN
Rosa26mTmG/+
Rosa26mTmG/Acrv1-iCre
Stage V
Stage VI
Stage IX
Stage XII-I

Fig. S2

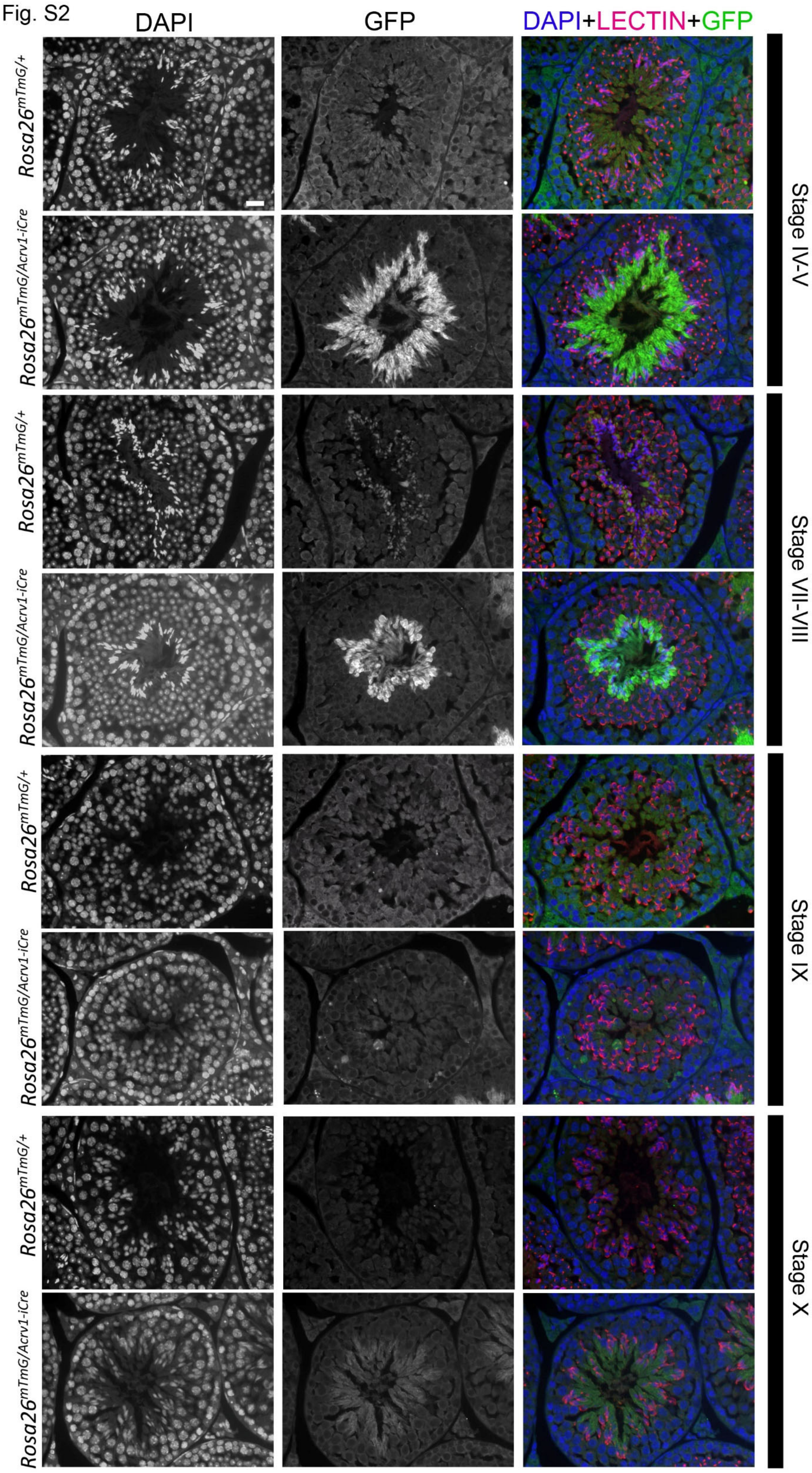

DAPI
GFP
DAPI+LECTIN+GFP
Rosa26mTmG/+
Rosa26mTmG/Acrv1-iCre
Stage IV-V
Stage VII-VIII
Stage IX
Stage X
